# Synergistic Effects of Laser-synthesized Silver Nanoparticles and Photosensitizers for enhanced Antibacterial and Anticancer activity

**DOI:** 10.1038/s41598-026-49267-8

**Published:** 2026-05-02

**Authors:** Saira Israr, Irfa Zafeer, Ifrah Shafqat, Ghulam Aisha, Aneela Javed, Shaista Shahzada, Shaheen Shahzad

**Affiliations:** 1 Genomics Research Lab, Department of Biological Sciences, International Islamic University, Islamabad, Pakistan; 2 Laser and Photonic Materials Laboratory, Department of Physics, International Islamic University, Islamabad, Pakistan; 3 Department of Biomedicine, Atta Ur Rehman School of Applied Biosciences National University of Sciences and Technology, Islamabad, Pakistan

**Keywords:** Gram-negative bacteria, Photodynamic therapy (PDT), Photosensitizer Chlorine e6 (Ce6) and Methylene blue (MB), Silver nanoparticles (AgNPs), Biochemistry, Biotechnology, Cancer, Chemistry, Drug discovery, Microbiology, Nanoscience and technology

## Abstract

The synergistic use of silver nanoparticles (AgNPs) and photosensitizer’s offers promise biomedical improvements. This study assesses and creates the potential for photosensitizers (Chlorine e6 (Ce6), Methylene Blue (MB)) and Silver Nanoparticles to work together to enhance biological activity. AgNPs were created by the laser ablation method and characterized using methods including scanning electron microscopy (SEM), Fourier transform infrared (FTIR) spectroscopy, and X-ray diffraction (XRD).The antibacterial and anticancer properties of these nanoparticles, both individually and in combination with photosensitizers, were further examined. AgNPs were combined with Methylene Blue and Chlorine e6 to enhance their antibacterial activity against Gram-negative bacteria, such as *Salmonella enteritidis*,* Pseudomonas aeruginosa*, and *Acinetobacter baumannii*, resulting in inhibition zones of up to as large as 0.66 ± 057 mm. The anticancer properties of the combination therapy were also examined against MCF-7 breast cancer cells, where Chlorine e6 alone had an IC50 of approximately 231.2%. Another photosensitizer, Methylene blue, showed a dose-dependent reduction in cell viability, with an IC50 of around 6.52 ± 3.26%. When AgNPs and Methylene Blue combined, the IC50 decreased to 11.42 ± 5.71, indicating a synergistic increase in cytotoxicity. Similarly, Chlorine e6 and AgNPs together significantly decreased the IC50 to 80µM to 100 µM. These findings show that the combined use of Methylene Blue or Chlorine e6 with AgNPs greatly improves anticancer and antibacterial efficacy compared to their individual applications. This research highlights how AgNPs and photosensitizers have the ability to change treatment approaches by providing improved specificity and efficacy in biomedical applications.

## Introduction

One of the most important problems facing modern public health is antibiotic resistance, which affects the effectiveness of current illness therapies. In the US, antibiotic-resistant diseases impact around three million people each year and cause more than 35,000 deaths, according to a report from the Centers for Disease Control and Prevention (CDC). There is an urgent need for alternative antimicrobial techniques as resistant types of bacteria and fungus continue to spread throughout the world through people, animals, and products. Photodynamic treatment (PDT), metals, gene editing enzymes, phages, antimicrobial peptides, and other promising alternatives provide innovative ways to fight antibiotic resistance without creating new resistant strains^1^. Specifically, PDT generated interest because it can eliminate antibiotic-resistant bacteria by using light-activated photosensitizers (PS), which produce reactive oxygen species (ROS) that kill dangerous cells without increasing resistance.

A number of alternative anti-microbial technologies, such as photodynamic therapy (PDT), metallic nanoparticles, antimicrobial peptides, phages, and gene editing enzymes, have been investigated by researchers to address this problem^2–4^.The non-invasive, light-dependent technique of PDT, which uses a photosensitiser (PS) to produce reactive oxygen species (ROS) when exposed to particular light wavelengths, has garnered increasing attention among these. These ROS molecules cause cell death without the possibility of genetic resistance accumulation by upsetting vital biological components.

Through localised activation of photosensitisers, photodynamic therapy (PDT) treatment can target tumour cells as well as infectious organisms. Its broad-spectrum activity against viruses, bacteria, and fungi combined with its quick action—usually within hours as opposed to days—makes it effective. When compared to traditional antibiotics, PDT is a better strategy^5^. Additionally, because PDT targets several intracellular targets at once, resistance development and microbial adaptation are still very uncommon^6^.

High singlet oxygen yield, robust absorption in the red to near-infrared (NIR) spectrum, outstanding photo stability, and little dark toxicity are all necessary for a good photosensitiser^7^. Due to their effective ROS generation and absorption maxima in the 650–700 nm range, methylene blue and chlorine e6 (Ce6) have demonstrated remarkable promise among the available chemicals^8–10^. Both substances show great promise in anti-cancer applications and strong antibacterial activity against both gramme positive and gramme negative bacteria.

The synergistic potential of metalic nanoparticles, especially silver nanoparticles (AgNPs), when paired with photosensitizers has been highlighted by recent developments in nanomedicine. By releasing Ag+ ions, which damage bacterial membranes and proteins, AgNPs have intrinsic antibacterial action^2,6,11,12^. Furthermore, AgNPs have surface Plasmon resonance (SPR) characteristics that strengthen neighboring photosensitizers’ light absorption effectiveness by enhancing local electromagnetic fields. Increased ROS production and enhanced therapeutic efficacy under radiation are the results of this process.

AgNPs have been extensively investigated for their anticancer potential in oncology, mostly due to their capacity to cause oxidative stress, improve medication absorption, and increase PDT-mediated ROS generation^13^. Their physicochemical characteristics minimize off-target toxicity by enabling the targeted administration of photosensitisers to tumour tissue. In order to obtain increased cytotoxicity against both bacterial and cancer cells, AgNPs combined with Chlorine e6 and Methylene blue MB constitute a unique dual modality system that integrates photodynamic (PDT) and photothermal mechanism (PTT) processes.

The synergistic interaction of laser-synthesised AgNPs and photosensitisers under controlled irradiation has not been thoroughly studied^14,15^, despite earlier study on PDT or AgNPs alone. In order to close that gap, this study synthesises surfactant-free, pure AgNPs using pulsed laser ablation in liquid (PLAL) and evaluates them against gramme negative bacterial strains and MCF-7 breast cancer cells in combination with Ce6 and MB. This study is novel because it shows how light-triggered synergism can improve ROS-mediated cytotoxicity and antibacterial activity using laser-fabricated AgNPs devoid of chemical impurities.

This study offers fresh insights into the dual antibacterial and anticancer applications of AgNP photosensitiser composites by connecting nanotechnology and photomedicine, providing a promising avenue for next-generation, resistance-free treatment approaches. Recent studies also highlight the role of eco-friendly and green-synthesized nanoparticles in improving antimicrobial and anticancer performance^16,17^.

## Materials and Methods

### List of chemicals

LB Nutrient Agar (Thermo Fisher Scientific), 0.9% Physiological Saline Solution, Distilled water and Deionized Water (DI)(LABCONCO), 80% ethanol Solution, Chlorine e6, Silver nanoparticles, Gram negative bacteria, (Salmonella, Pseudomonas, Acinetobactor, NaCl (DAEJUNG)LOT N0. S0119PK1, Whatman membrane filter, PBS (Phosphate buffered saline), DMSO (Dimethyl sulfoxide) (DUKSAN REAGENTS) LOT N0. L3P115, 70% Ethanol.

### Synthesis of silver nanoparticles

Surfactant-free spherical silver nanoparticles (NPs) are created using the Pulsed Laser Ablation in Liquid (PLAL) technology, a method widely recognized for producing high-purity nanoparticles without chemical contaminants^14,18^. A pure silver target was used to synthesize AgNPs. Ag NPs in aqueous solution were produced by directing a laser beam of fundamental wavelength 1064 nm using Nd: YAG laser (Quantel Q-Smart 850 mJ, 10 Hz repetition rate, and 6 ns pulse duration) onto the silver surface. Deionized water is used as a solvent. The bottom of a 250 ml beaker held the pure silver target (99.99%, thickness 1.0 mm, weighed 15 g). After that, the beaker was filled with 30 ml of deionized water, increasing the liquid’s height above the target by 11 mm. Then, the beaker was placed on the XY-translation stage to change the position of the laser beam with respect to the target. Moreover, this system was shaker-rotated at 312 rpm to minimize shielding effects and prevent Ag NPs accumulation above the target. After adjusting the laser beam’s energy to between 200 mJ, it focused using a converging lens with a focal length of 20 cm. The ablation process takes about 30 min. Synthesized Ag-NPs’ concentration was calculated using inductively coupled plasma optical emission spectroscopy (ICP-OES)^19^.

### Characterization of AgNPs

In order to assess the morphology, size, and optical characteristics of the produced nanoparticles, characterization was done using UV-Vis spectroscopy, Fourier-transform infrared spectroscopy (FTIR), scanning electron microscopy (SEM), and X-ray diffraction (XRD)^20,21^. Silver nanoparticles (AgNPs) were detected by measuring their absorbance in various solutions using UV-Vis spectrophotometry, and their presence was verified by surface plasmon resonance (SPR) peaks. SEM verified the composition of the AgNPs by revealing information on their shape, microstructure, and size distribution^22^. By using Scherrer’s equation to the peak broadening of the diffraction plane, the average crystallite size was determined from the XRD pattern^23^. FTIR is an efficient tool for examining material surfaces and bulk properties up to 11 mm in diameter since it can be used for both qualitative identification and quantitative analysis when standards are applied^24^.

### Assessment of antimicrobial activity of Ag NPs and chlorine e6 (Ce6) using photodynamic therapy

Using the well diffusion method, the antibacterial activity of silver nanoparticles (AgNPs) produced by laser ablation technology was assessed against *Salmonella enteritidis*,* Pseudomonas aeruginosa*, and *Acinetobacter baumannii*^11^. Lyophilized chlorin e6 trisodium salt (Ce6), of high purity (~ 98%), was handled in a dark environment to avoid light exposure. A stock solution was prepared by dissolving 0.006 g of Ce6 in 10 mL of double-deionized sterile water (ddH₂O) and stored at -20 °C. Working concentrations of 100–200 µM were freshly prepared in sterile physiological saline (0.9% NaCl, pH 7.4) before use^25^.

Bacterial cultures were incubated in nutrient agar, and four experimental groups were set up: (1) bacteria alone, (2) bacteria + Ce6, (3) bacteria + AgNPs, and (4) bacteria + AgNPs + Ce6, each in duplicate. One sample from each group was exposed to red light (650–700 nm) for 15–20 min, while the other remained as a control^26^. Photodynamic therapy (PDT) using Ce6, combined with AgNPs, significantly reduced bacterial counts. The highest concentration of Ce6 that was bactericidal was 100 µM. Samples were incubated at 37 °C for 24 h following light exposure, and the results showed a significant reduction in bacterial colonies, indicating the AgNPs and Ce6 combination’s improved antibacterial activity under PDT conditions^6^.

The combined system can still function well under a single irradiation situation because of the following factors, even though Ce6 and methylene blue (PDT agents) show maximum absorption around 660 nm and silver nanoparticles (PTT agents) display peak photothermal activity near 880 nm.

First, even when the irradiation wavelength does not precisely match each of Ce6’s and AgNPs’ individual maxima, partial excitation is still possible due to their comparatively wide absorption spectra that reach into the near-infrared (NIR) region. Second, by increasing oxygen transport and improving cellular uptake, the localised photothermal effect produced by AgNPs can boost the photodynamic efficiency of Ce6 or methylene blue.

Furthermore, an intermediate or wide NIR light source (usually within 650–800 nm) is used in many PDT–PTT combination systems to simultaneously activate both components, resulting in a synergistic therapeutic response. Therefore, the Ce6/MB–AgNPs system may function well under the same irradiation conditions despite the discrepancies in their optimal activation wavelengths due to the overlapping spectrum features and photothermal–photodynamic synergy.

### Assessment of antimicrobial activity of Ag NPs and Methylene blue using photodynamic therapy

Methylene blue (MB) antibacterial activity was assessed by preparing solutions with varying concentrations using distilled water. In 5 ml of distilled water, 0.0097 g of MB (Sigma Aldrich) were dissolved to create a stock solution with a molarity of 6100µM. Then, using this stock, dilutions of 610 µM, 61 µMM, and 6.1 µM were made in a final volume of 1 mL. *Acinetobacter baumannii*,* Pseudomonas aeruginosa*, and *Salmonella enteritidis* were among the Gram-negative bacteria that were used to investigate the antibacterial activity of silver nanoparticles (AgNPs) in combination with MB. Bacterial suspensions were made after bacterial cultures were grown in nutrient agar medium for 18 to 24 h. Using a red light source with a wavelength of 650–700 nm, photodynamic treatment (PDT) was used.The experiment was divided into four groups: (1) bacteria alone, (2) bacteria + MB, (3) bacteria + AgNPs, and (4) bacteria + AgNPs + MB, each prepared in duplicate. One set from each group was exposed to red light for 15–20 min, while the other served as a control. After incubation at 37 °C for 24 h, bacterial counts were assessed to evaluate the antimicrobial effects^1,6^.

### MTT (3-(4, 5-dimethylthiazol-2-yl)-2, 5-diphenyltetrazolium bromide) assay for anticancer activity

The MTT test used to evaluate the cytotoxicity of mixed silver nanoparticles with Ce6 and MB (a photosensitizer) and Chlorine e6, Methylene blue against a human breast cancer (MCF-7) cell line using photodynamic treatment chemicals. The MCF-7 (ATCC HTB-22) cell line was kindly provided by Dr. Rumeza Hanif, Associate Professor at the Atta-ur-Rahman School of Applied Biosciences (ASAB), NUST, Islamabad. The cell line was originally purchased from ATCC and preserved in the cell culture bank at ASAB. Cells were cultured for cell line activity in Dulbecco modified eagle media (DMEM)–high glucose with 10% FBS (Thermo Fischer Scientific, Waltham, MA, USA) and 1% pen-strep MTT (3-[4, 5-dimethylthiazol-2-yl]-2,5-diphenyltetrazolium bromide (Sigma-Aldrich, St. Louis, MO, USA). Exponentially growing cells (10,000 cells per well) were plated, in triplicate, in flat-bottomed 96-well plates (Nunc, Roskilde, Denmark). After the cells were adherent 24 h after plating, 100 µl of test compounds at different doses (80 µM/ml, 100, 150, and 200 µM/ml) Ce6 and (6100 µM to 6.1 µM) MB were given to the cells. The cells were then cultured for 48 h at 37 °C in an incubator.After incubation, 5 mg mL-1 of MTT was dissolved in 1 mL PBS and 15 µL of prepared MTT solution was added to each well and incubated for 3 h at 37 °C. Following the formation of formazan crystals, all the solution from each well was removed. Solubilizing solution (DMSO) was added to each well. The plates were left at room temperature for few min. The absorbance of the cells was recorded at 550 nm. Experiments were performed in triplicates^27^. This study focused exclusively on evaluating the cytotoxic response of MCF-7 cancer cells to assess the potential anticancer effect of the synthesized nanoparticles. Cytotoxicity toward normal cells was not included in the present experimental design and will be addressed in future biocompatibility studies. It is generally reported in PDT-based therapies that non-irradiated areas remain less affected, minimizing off-target toxicity toward normal tissues.

### Photodynamic effects (PDT)

The cells were grown in a 3.4 cm petri dish, with the red light spot size covering the entire cell monolayer. The light source was positioned 8 cm above the cells. For treatment, red light lasers at a wavelength of 660 nm, which is the excitation range for the Ce6 and MB photosensitizer, were used. The cells were categorized into two groups: Group 1 consisted of cells treated with Ce6 and MB alone, while Group 2 included cells treated with both Ce6, MB and Ag NPs^28,29^.

## Results

### Characterization of synthesized AgNPs

This study focuses on the synthesis of silver nanoparticles using pure silver through laser-based methods, with detailed findings presented in the report. Silver nanoparticles were created from the silver ions. After 30 min and an hour of the reaction, it was noticed that the color of the solution changed from yellow to brilliant yellow and finally to dark brown, indicating the creation of silver nanoparticles.

#### UV-vis analysis

The optical properties of silver nanoparticles and their oxides were examined using UV-Vis spectrophotometry. Silver nanoparticles (AgNPs) were detected by measuring their absorbance in various solutions, and their presence was verified by distinct absorption peak near 410 nm (shown in Fig. 7), which corresponds to the surface plasmon resonance (SPR) of silver nanoparticles. This peak indicates small, spherical Ag nanoparticles. The sharpness and symmetry of the peak suggest the nanoparticles are uniform and well dispersed, with minimal aggregation. The relatively higher absorbance observed at shorter wavelengths suggests a greater concentration of synthesized nanoparticles in the solution. Beyond 450 nm, the low absorption further suggests the great strength and dispersion of the silver nanoparticles in the solution. Overall, the UV-Vis spectral analysis verifies the effective production of silver nanoparticles.

**Fig. 1:**
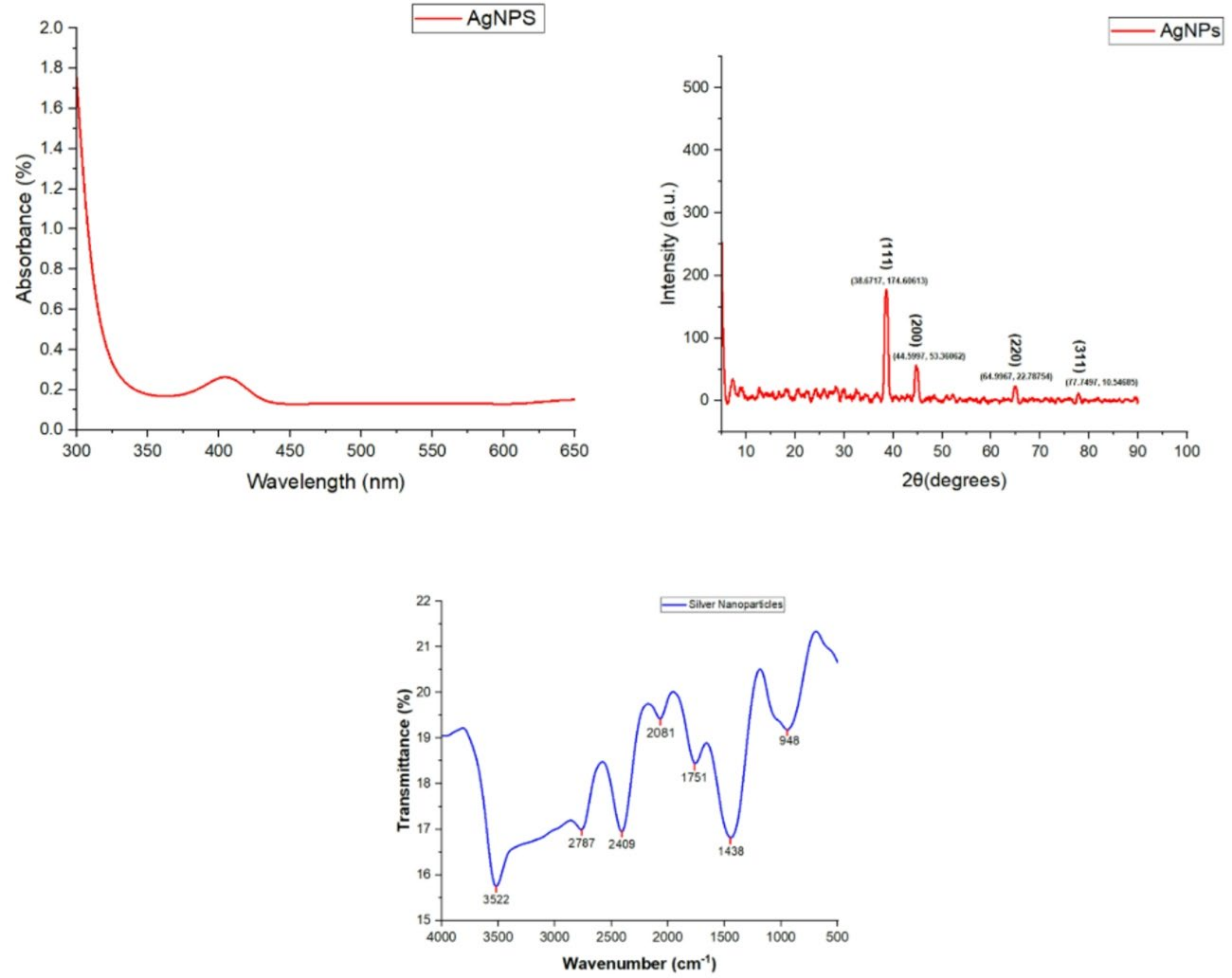
UV-visible spectroscopy, X-ray diffraction and FTIR spectra of laser ablated Ag NPs.

#### X-ray diffraction (XRD)

The crystalline nature of the synthesized silver nanoparticles was confirmed by X-ray diffraction (XRD) analysis. The XRD pattern (Fig. 7) displays distinct diffraction peaks which corresponds to different crystallographic planes of face-centered cubic (FCC) silver. The diffraction peaks observed at approximately 38.67^o^, 44.59 ^o^, 64.99 ^o^, and 77.29 ^o^ are indexed as (111), (200), (220), and (311) planes, respectively, according to the standard JCPDS card for silver (No. 04-0783). The intense reflection from (111) plane indicates that it is the most densely packed and preferentially oriented plane in the crystal structure. The sharp and well-defined nature of peaks confirms the high crystallinity and phase purity of synthesized silver nanoparticles. The grain size was determined using the Debye-Scherrer equation,

$$\:\mathrm{D}\:=\frac{\mathrm{K}{\uplambda\:}}{{\upbeta\:}\mathrm{c}\mathrm{o}\mathrm{s}{\uptheta\:}}$$

where constant K is called shape factor whose value is around 0.94, λ is the wavelength of x-ray which depends on the X-ray source; for example, for a standard copper K-alpha (CuKα) source, the wavelength is approximately 1.541 Å, β is the full width at half maximum (FWHM) in radians, whereas θ represent the peak position. Here the average crystallite size was found to be approximately 25 nm. The peaks’ existence and positions confirm the silver nanoparticles’ fresh, well-defined crystalline structure, which is characteristic of silver.

#### Fourier transform infrared spectroscopy (FTIR)

The biological activity of the synthesized AgNPs greatly depends on the functional groups identified in the FTIR spectrum. The broad band in the region of 3400–3500 cm⁻¹ exhibits O–H stretching vibrations of hydroxyl groups, which facilitate hydrogen bonding and stable nanoparticles in solution. C = O and C–O amide and carboxyl group stretches are represented by the peaks at approximately 1630–1750 cm⁻¹ as illustrated in Fig. 7, which point towards the presence of biomolecules that cap and reduce Ag⁺ ions during synthesis. By facilitating protein binding and interaction with the cell membrane, these surface functional groups stimulate the generation of reactive oxygen species (ROS) and facilitate the antibacterial and anticancer activity that has been reported.

#### Scanning electron microscopy (SEM)

The SEM images illustrates the surface morphology and particle size distribution of the synthesized silver nanoparticles. The image reveals nanoparticles with varied sizes and well-defined spherical morphology. The measured average diameters of selected nanoparticles D = 40 nm. These measurements indicate a heterogeneous size distribution, ranging from approximately 23 nm to 66 nm. The diversity in particle size could be attributed to the synthesis method or the conditions under which the nanoparticles were formed. It confirms the successful synthesis of silver nanoparticles with a relatively broad size distribution, which may impact their optical, chemical, and physical properties as illustrated in Fig. 8.

**Fig. 2:**
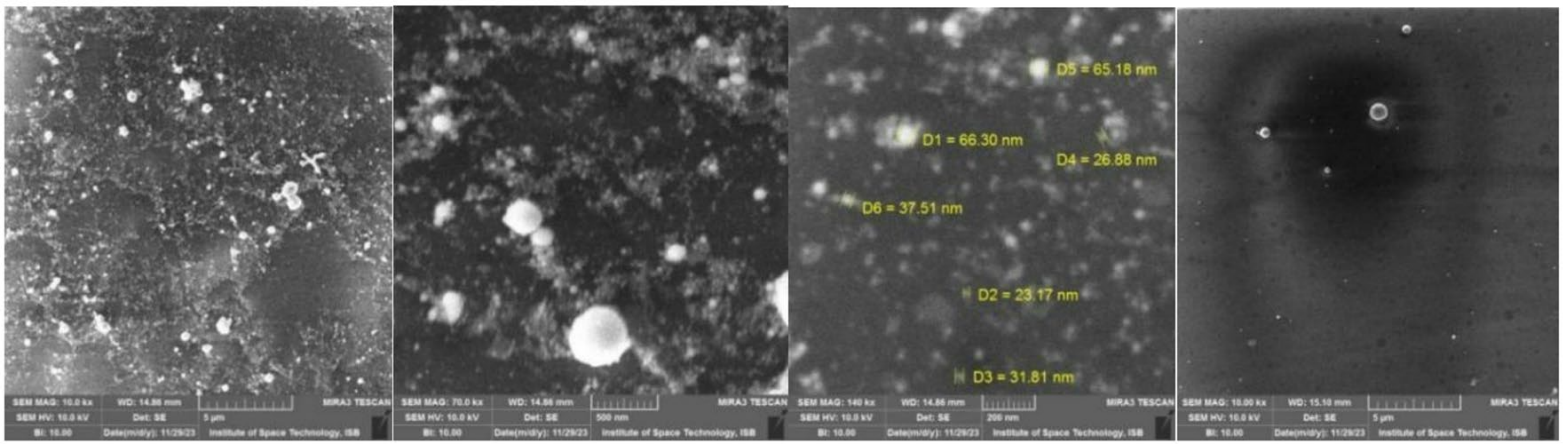
SEM micrograph of AgNPs synthesized from Laser Ablation method.

### Silver quantification by ICP OES

A linear connection between silver concentration (ppm) and signal intensity (a.u.) is demonstrated in Fig. 9, which shows the quantification of silver (Ag) using Inductively Coupled Plasma Optical Emission Spectroscopy (ICP-OES). The efficiency of ICP-OES for precisely detecting silver concentrations is shown by measurements taken at 15 and 45 min, which show that when the concentration of silver increases, the detected signal’s intensity likewise increases proportionately. The accuracy of ICP-OES in measuring silver in different samples is validated by this linear trend (Fig. 9).

**Fig. 3:**
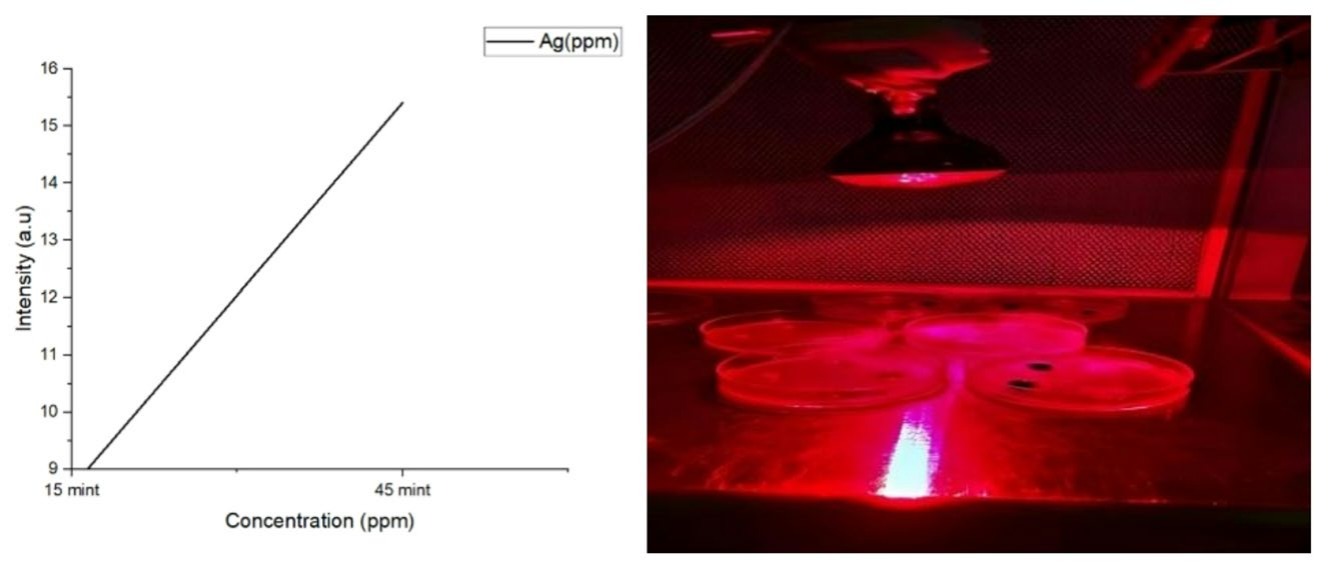
Silver quantification by ICP OES & Exposure of samples with red light.

### Antibacterial activity

Table 1 displays the zone of inhibition for silver nanoparticles (Ag NPs) produced by laser ablation method and evaluated at three distinct time intervals: 15 min, 20 min, and 45 min, against *Pseudomonas aeruginosa*,* Salmonella enteritidis*, and *Acinetobacter baumannii* (Fig. 10).

**Table 1:** Zone of inhibition of Silver Nanoparticles Synthesized by Laser Ablation Method (Lack of observable inhibitory regions on control plates with bacteria free of silver nanoparticles proved that antibacterial activity depended solely on the presence of silver nanoparticles (AgNPs).

Zone of inhibition of silver nanoparticles									
*Pseudomonas aeruginosa*				*Salmonella enteritidis*			*Acinetobactor baumannii*		
Time (min)	15 Min	20 Min	45 Min	15 Min	20 Min	45 Min	15 Min	20 Min	45 Min
Avr. /SE	0.66 ± 0.57	0.66 ± 0.57	0.66 ± 0.57	0.66 ± 0.57	0.66 ± 0.57	0.44 ± 0.33	0.44 ± 0.33	0.66 ± 0.57	0.66 ± 0.57

**Fig. 4:**
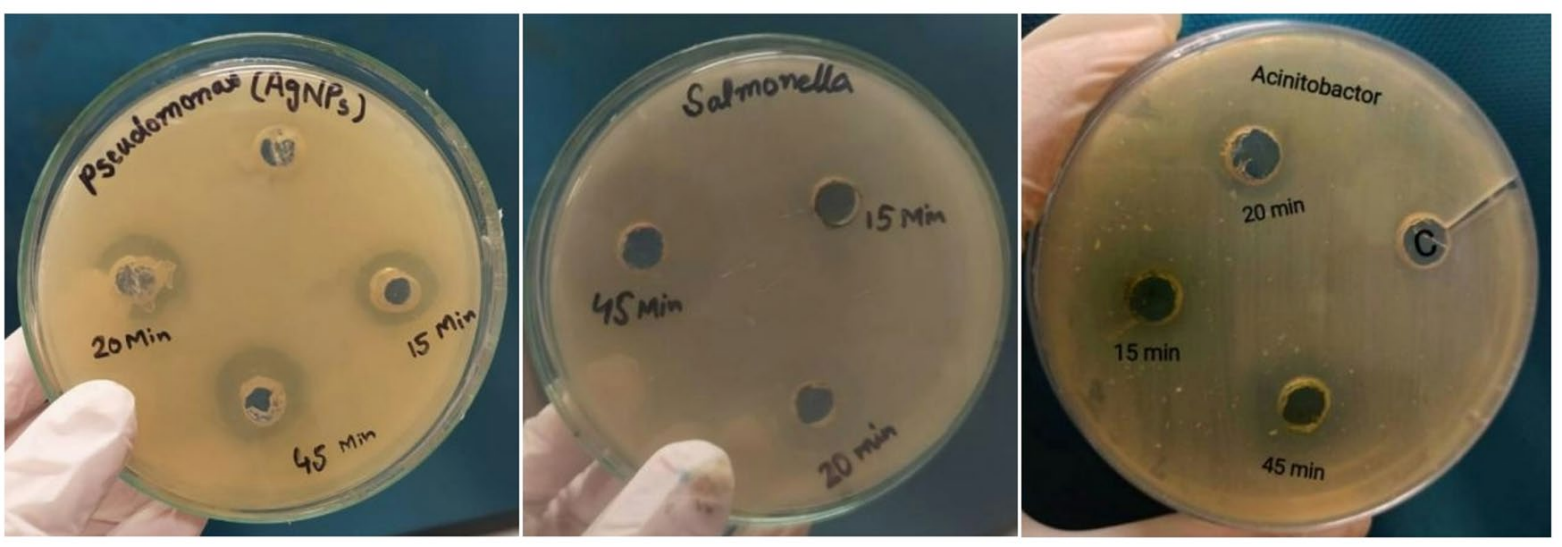
Zone of inhibition of silver nanoparticles against various pathogenic bacteria.

#### Synergistic effect of silver nanoparticles combined with Chlorine e6 and Chlorine e6 alone

Table 2 summarizes the antibacterial efficacy of Chlorine e6 and silver nanoparticles (AgNPs) against *Pseudomonas aeruginosa*,* Salmonella enteritidis*,* and Acinetobacter baumannii* by detailing their zones of inhibition. The highest zone of inhibition recorded was 1 ± 0.57 mm for *Pseudomonas aeruginosa* at a concentration of 200 µM when treated with AgNPs, indicating significant antibacterial activity at elevated doses. In contrast, the lowest zone of inhibition was observed for *Salmonella enteritidis* at 150 µM, where only a minimal inhibition of 0.44 ± 0.33 mm was recorded. This stark difference highlights the effectiveness of combining AgNPs with Chlorine e6 to enhance antibacterial activity, particularly against resistant bacterial strains (Fig. 11).

**Table 2:** Zone of inhibition of Silver nanoparticles combined with Chlorine e6 photosensitizer and Chlorine e6 alone. Negative controls, or the bacteria that did not have Ce6 or AgNPs, showed normal growth in all experiments, verifying that the inhibitory zones resulted only from the synergistic effect of AgNPs and Ce6 under photodynamic therapy. The data are reported as the mean ± SD of three independent tests.

	Chlorine e6 (Ce6) (µM)					Ag NPs + Ce6 (µM)			
		80	100	150	200	80	100	150	200
*Pseudomonas aeruginosa*	AVG/SE	0.44 ± 0.33	0.44 ± 0.33	0.44 ± 0.33	0.66 ± 0.57	0.66 ± 0.57	0.44 ± 0.33	0.44± 0.33	0.44 ± 0.33
*Salmonella enteritidis*	AVG/SE	1.33 ± 0.99	0.66 ± 0.57	0.44 ± 0.33	0.66 ± 0.57	0.44 ±0.33	1.11± 0.87	0.66± 0.57	2.66 ± 2.07
*Acinetobactor baumannii*	AVG/SE	0 ± 0	0.44 ± 0.33	0.44 ± 0.33	0 ± 0	0.44 ± 0.33	0.66± 0.57	0.66 ± 0.57	0.44 ± 0.33

**Fig. 5:**
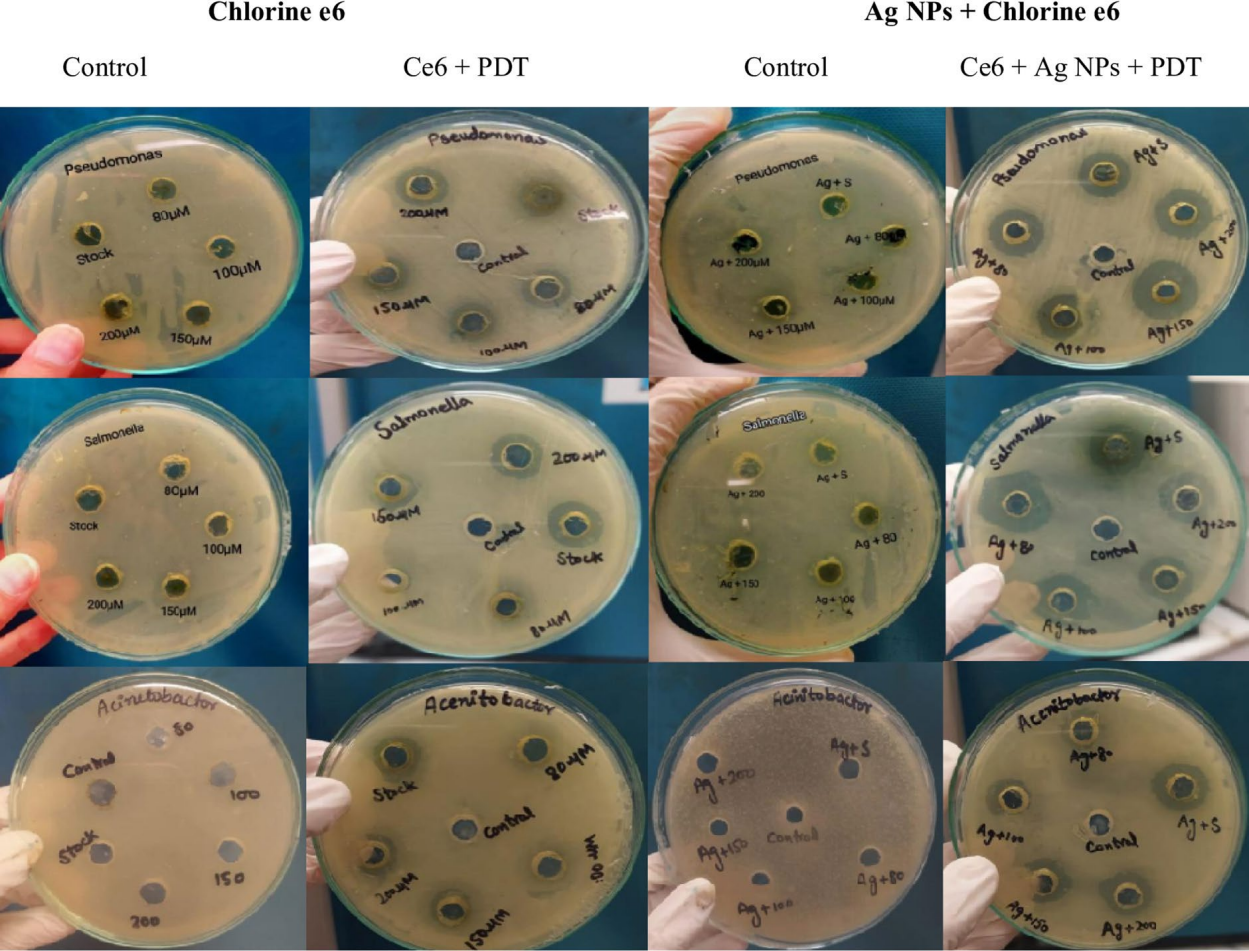
Zone of inhibition of *Pseudomonas aeruginosa*,* Salmonella enteritidis*,* Acinetobactor baumannii*.

#### Exposure of samples with red light

Visual evidence of photodynamic treatment (PDT) uses petri dishes exposed to red light (wavelength: 600–700 nm) is presented. Clear zones of inhibition are created by this therapy by using light to activate a photosensitizer, which subsequently kills bacteria. *Pseudomonas aeruginosa*,* Salmonella*, and *Acinetobacter baumannii* are among the several Gram-negative bacteria that are present in the petri dishes. There were zones of inhibition as a result of the photosensitizer killing the bacteria following the light therapy. In the absence of photodynamic treatment, no zones of inhibition were observed, demonstrating that light is required for the photosensitizer activation to be effective against these bacteria. Various PDT-prepared samples were subjected to different durations of red-light exposure.

#### Synergistic effect of silver nanoparticles combined with methylene blue and methylene blue alone

**Fig. 6:**
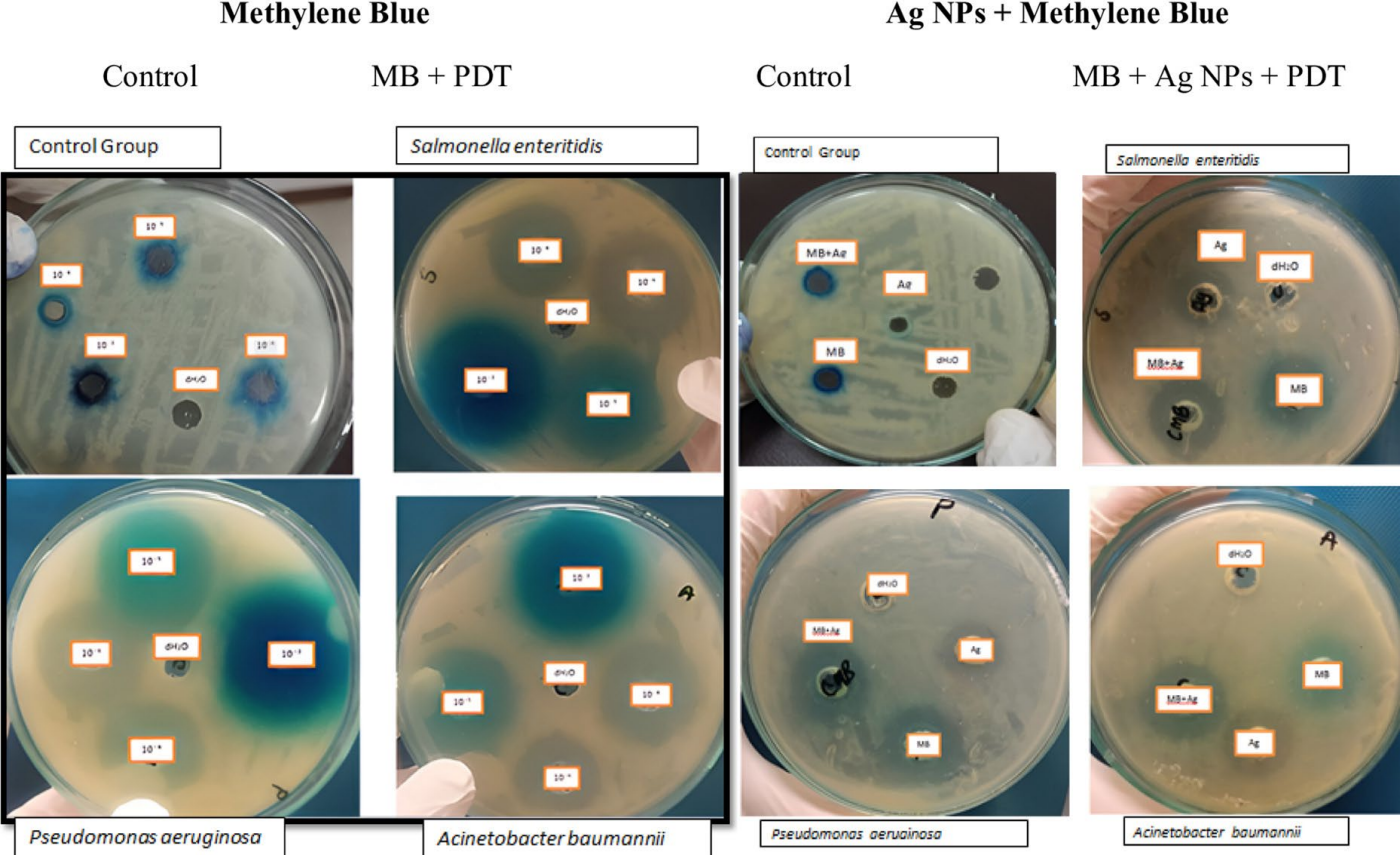
Effect of methylene blue concentrations with light and without light on bacterial growth, & effect of methylene blue concentration, silver nanoparticles and combine MB and Ag with light and without light on bacterial growth.

Particularly in photodynamic settings, the data analysis reveals strain-specific reactions to different concentrations of Methylene Blue (MB), silver nanoparticles (AgNPs), and their combination as shown in Table 3. With negligible inhibitory zones at all concentrations and no improved effect when paired with AgNPs, *Salmonella enteritidis* showed limited susceptibility, with the greatest bacterial growth recorded at 610 µM MB. However, the overall antibacterial action remained small and varied, with *Pseudomonas aeruginosa* exhibiting slightly higher inhibition at lower MB doses, peaking at 6.1 µM (0.13 mm). Inconsistent responses were shown by *Acinetobacter baumannii* the highest inhibition was recorded with MB alone (0.13 mm), outperforming both AgNPs alone and the combination therapy, suggesting that MB may be more effective on its own against this strain. The effectiveness of the therapies was confirmed by the control groups’ lack of antibacterial activity. High standard deviations indicated significant variability across all strains, highlighting the necessity of improved procedures or more duplicates to guarantee consistency and dependability when evaluating antimicrobial efficacy (Fig. 6).

**Table 3:** Zone of inhibition of Silver Nanoparticles combined with methylene blue photosensitizer and methylene blue alone. The synergistic PDT effect was accountable for the antibacterial activity seen, as indicated by an absence of inhibitory zones in control groups consisting of bacterial cultures to which light, AgNPs, or Methylene Blue had not been applied. The variation in zone size is illustrated as mean ± SD for triplicate trials.

	Methylene blue (µM)					Ag NPs + methylene blue			
		6100	610	61	6.1	Control	Ag NPs	MB	Ag NPs + MB
*Pseudomonas aeruginosa*	AVR	0 ± 0.02	0.08 ± 0.06	0.08 ± 0.06	0.13 ± 0.09	0 ± 0	0.04 ± 0.02	0.06 ± 0.05	0.06 ± 0.05
*Salmonella enteritidis*	AVR	0.06 ± 0.05	0.08 ± 0.06	0.04 ± 0.02	0.04 ± 0.02	0 ± 0	0.04 ± 0.02	0.06 ± 0.05	0 ± 0
*Acinetobactor baumannii*	AVR	0.11 ± 0.08	0.11 ± 0.08	0.06 ± 0.05	0.11 ± 0.08	0 ± 0	0 ± 0	0.13 ± 0.11	0 ± 0

### Cytotoxicity assay

The anticancer potential of Silver Nanoparticles alone and with combined Ce6 and MB were screened against breast cancer MCF-7 cell line (Table 4). A dose dependent anti-cancer response was noticed with rising concentration of the test compounds, sharp reduction in the viability percentage of the cells were noticed. The cell viability percentages indicate the proportion of living cells out of the total cells present. For instance, at a Chlorine e6 concentration of 200 µM/mL, the cell viability is 33.75%, meaning that 33.75% of the cells survive at this concentration. Similarly, at Chlorine e6 concentrations of 150 µM/mL, 100 µM/mL, and 80 µM/mL, the viability percentages are 39.35%, 84.57%, and 88.05%, respectively as illustrated in fig. 14.20. In case of Methylene blue the concentration of MB necessary to block 50% of cell viability: 22.72163%, 33.3747%, 34.82697% and 47.3735% are the values for 6100µM, 610 µM, 61 µM and 6.1 µM. This demonstrates that as the Chlorine e6 and Methylene blue concentration decreases, a greater proportion of cells survive, reflecting a dose-dependent reduction in cell viability with higher photosensitiser concentrations. Different concentrations (80µM, 100µM, 150 µM and 200 µM)of Chlorine e6, (6100µM, 610µM, 61µM and 6.1 µM) of Methylene blue and (80µM/ml + Ag NPs, 100µM/ml + Ag NPs, 150 µM /ml + Ag NPs and 200 µM /ml + Ag NPs) (6100µM, 610µM, 61µM and 6.1 µM + Ag NPs and 6.1 µM + Ag NPs) of Synthesized Silver Nanoparticles were used to investigate the growth inhibition of cancer cells in human breast cancer cell line (MCF-7). To ascertain the reduction in cancer cell viability brought on by cytotoxic drugs, the MTT [3- (4,5-dimethylthiazol-2-yl)-2,5-diphenyltetrazolium bromide] assay was utilized. For Human Breast Cancer, the IC50 value, which is the drug concentration needed to achieve 50% inhibition of cell viability in human breast cancer cells, was found to be 138.2 µM/mL for Chlorine e6 (Ce6) & 4.92 µM for Methylene blue (MB).

Untreated MCF-7 cells served as a negative control in all cytotoxicity assays; these cells maintained nearly 100% vitality after 48 h, proving that the decreases in the cell viability observed were only due to treatment. Each experiment was performed thrice, and data are presented as mean ± SD.

**Table 4:** MCF-7 cells treated with silver nanoparticles, Chlorine e6 (Ce6) and Chlorine e6(Ce6) combined with silver nanoparticles (Ag NPs) for 48 h.

	Concentration µg/mL	Cell viability %
Ag NPs	15.40	31.76
	10.25	43.25
	8.68	57.45
Chlorine e6 (Ce6)	200	33.75
	150	39.35
	100	84.57
	80	88.05
Chlorine e6 + Ag NPs	200	24.51
	150	25.83
	100	26.24
	80	28.78

#### MCF-7 cells treated with silver nanoparticles

**Fig. 7:**
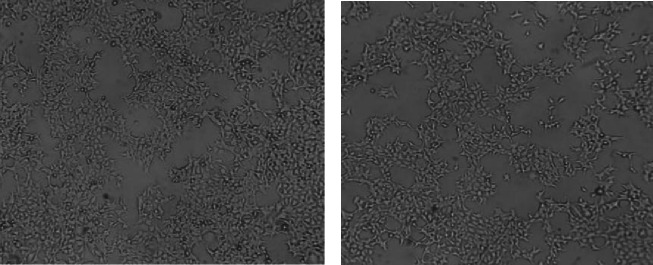
Non treated MCF-7 cells

**Fig. 8:**
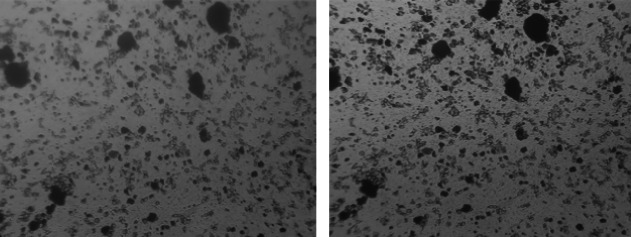
MCF-7 cells treated with Silver Nanoparticles for 48 hours.

The MTT test was utilized to assess the inhibitory impact of three distinct doses (15.40 µg/ml, 10.25 µg/ml, and 8.68 µg/ml) of AgNP on MCF-7 cell lines during a 48-hour incubation period. An IC50 value of around 9.50 µg/ml was computed, cell viability over doses ranging from 1 to 16 µg/m was produced. The findings unambiguously show that greater AgNP concentrations have a more cytotoxic impact. After 48 h of treatment, the average biomass of MCF-7 cells decreases as the concentration of AgNP increases, as per the colorimetric technique. Silver nanoparticles (Ag NPs) at concentrations of 15.40, 10.25, and 8.68 µg/mL showed an average cell viability of 31.76% ±57.45%. These results indicate high variability in cell response to Ag NP treatment (Figs. 7 and 8).

#### MCF-7 cells treated with Chlorine e6 (Ce6) for 48 h

Chlorine e6 (Ce6) was treated for 48 h on MCF-7 cells, a commonly utilized human breast cancer cell line, in order to assess any potential therapeutic benefits. Because it combines light with a photosensitizing chemical to form reactive oxygen species that can cause cell death. The 48-hour treatment interval gives Ce6 enough time to enter the MCF-7 cells and start working. Assessing the viability, proliferation, and morphological changes of cells after therapy might yield important information on how well Ce6 targets and inhibits the development of cancer cells.

**Fig. 9:**
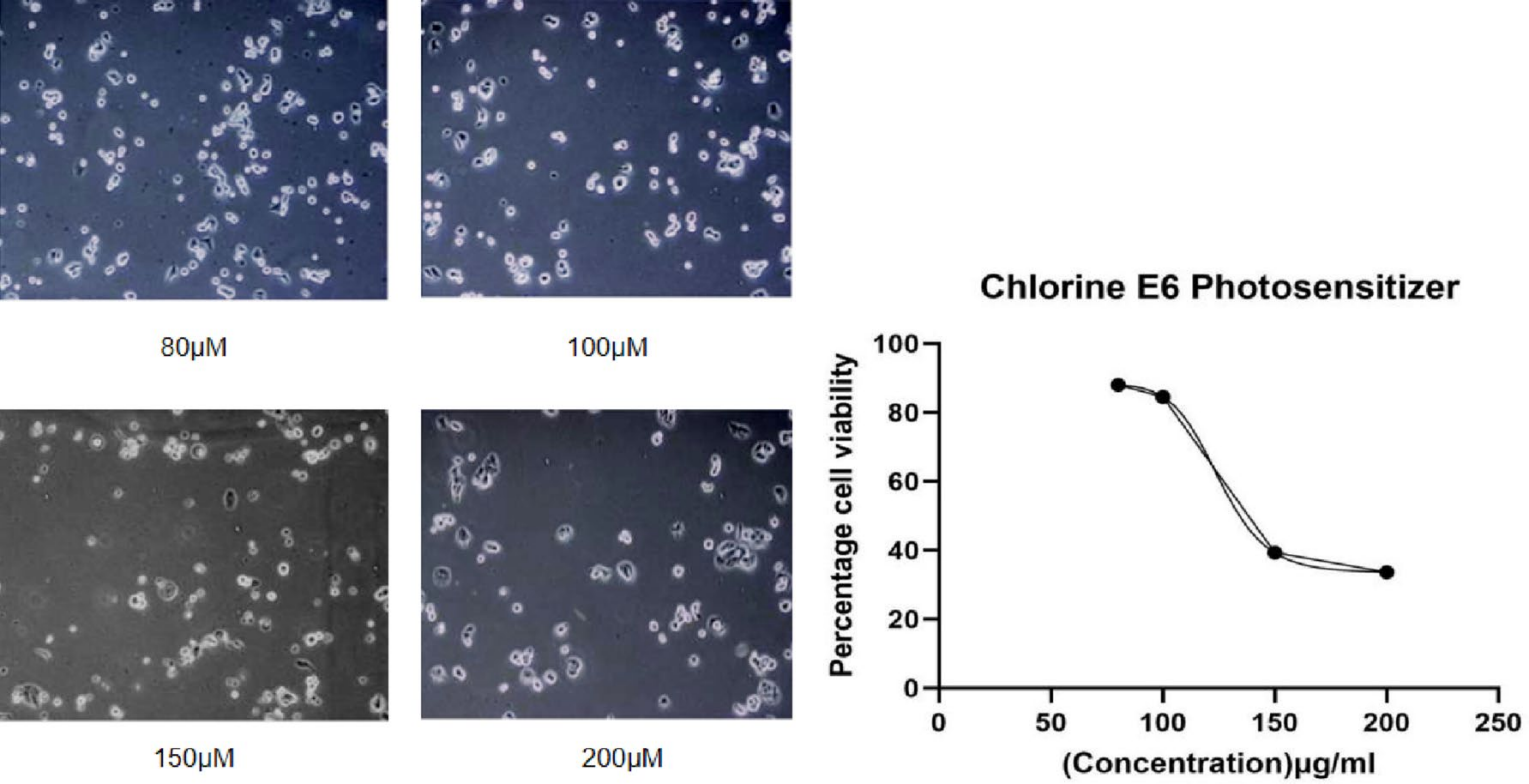
MCF-7 cells treated with 80, 100, 150 and 200 µM of Chlorine e6 (Ce6) for 48 hours.

In order to calculate the 50% inhibition of cell viability at a concentration (IC50), we carried out linear interpolation between concentrations at which cell viability was 50% and higher. With cell viability being below 50% at 150 µM/mL and over 50% at 100 µM/mL, the IC50 value falls between 100 µM/mL and 150 µM/mL. Therefore, the estimated IC50 value is approximately 138.2 µg/mL (Fig. 9).

#### Chlorine E6 photosensitizer combined with silver nanoparticles

**Fig. 10:**
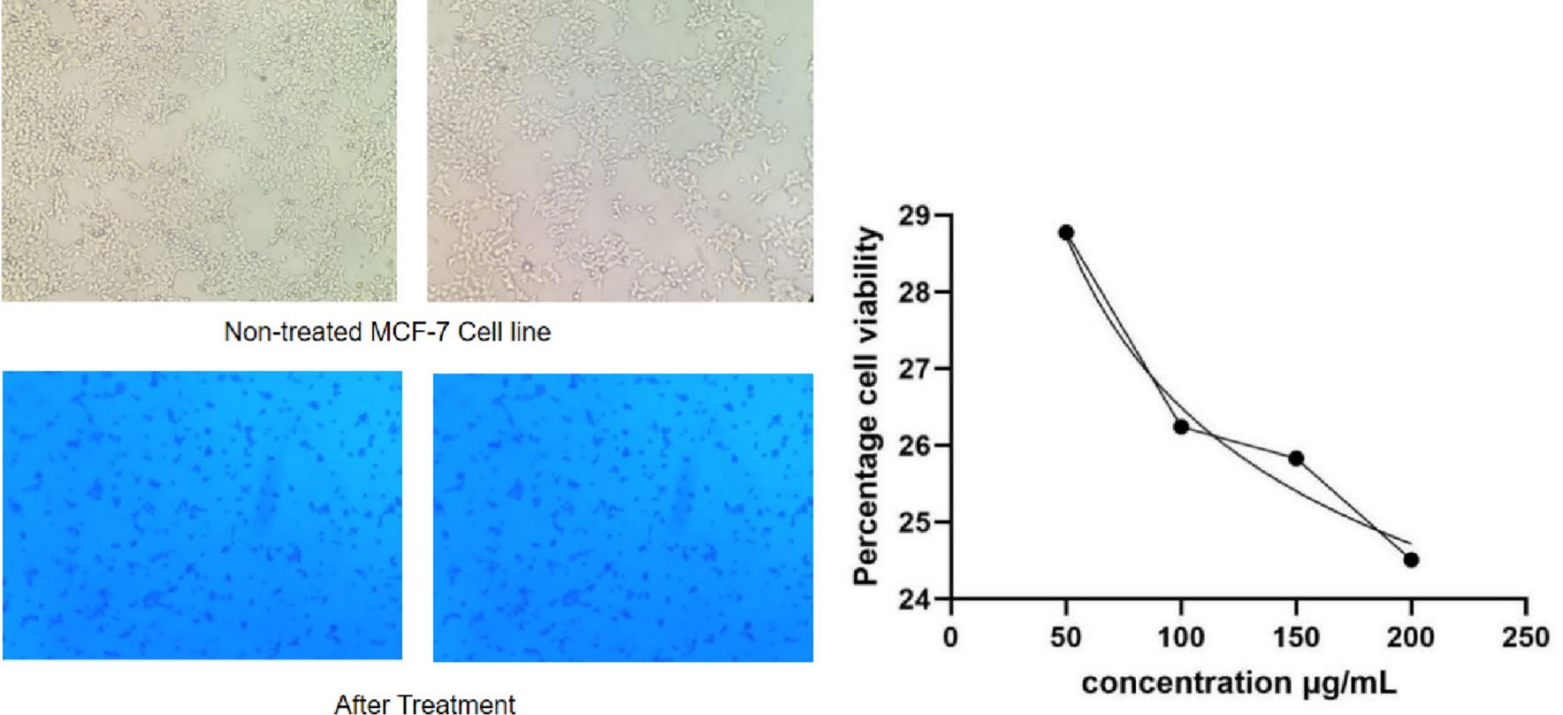
A magnified view (200×) of MCF-7 cells treated with Chlorine e6(Ce6) combined with Silver Nanoparticles (Ag NPs) for 48 hours demonstrates noticeable cellular shrinkage in the treated cells, as highlighted by the arrows.

When Chlorine E6 Photosensitizer is used in combination with Silver Nanoparticles, the data illustrates how different Chlorine e6 concentrations in combination with Ag NPs affect the viability of cells. Average Cell viability 1.22 ± 0.61%, respectively, at the doses of 200 µM/mL and 150 µM/mL, suggesting a potent cytotoxic impact. According to (Fig. 10) this tendency, decreasing the concentration of Ag NPs and Chlorine e6 has a little but beneficial impact on cell survival, but their presence significantly reduces cell viability. Since cell viability is lower than 50% at all tested concentrations, the IC50 is expected to be between 80 and 100 µM/mL.

#### Combined action of MB and silver NPs on cancer cells

**Fig. 11:**
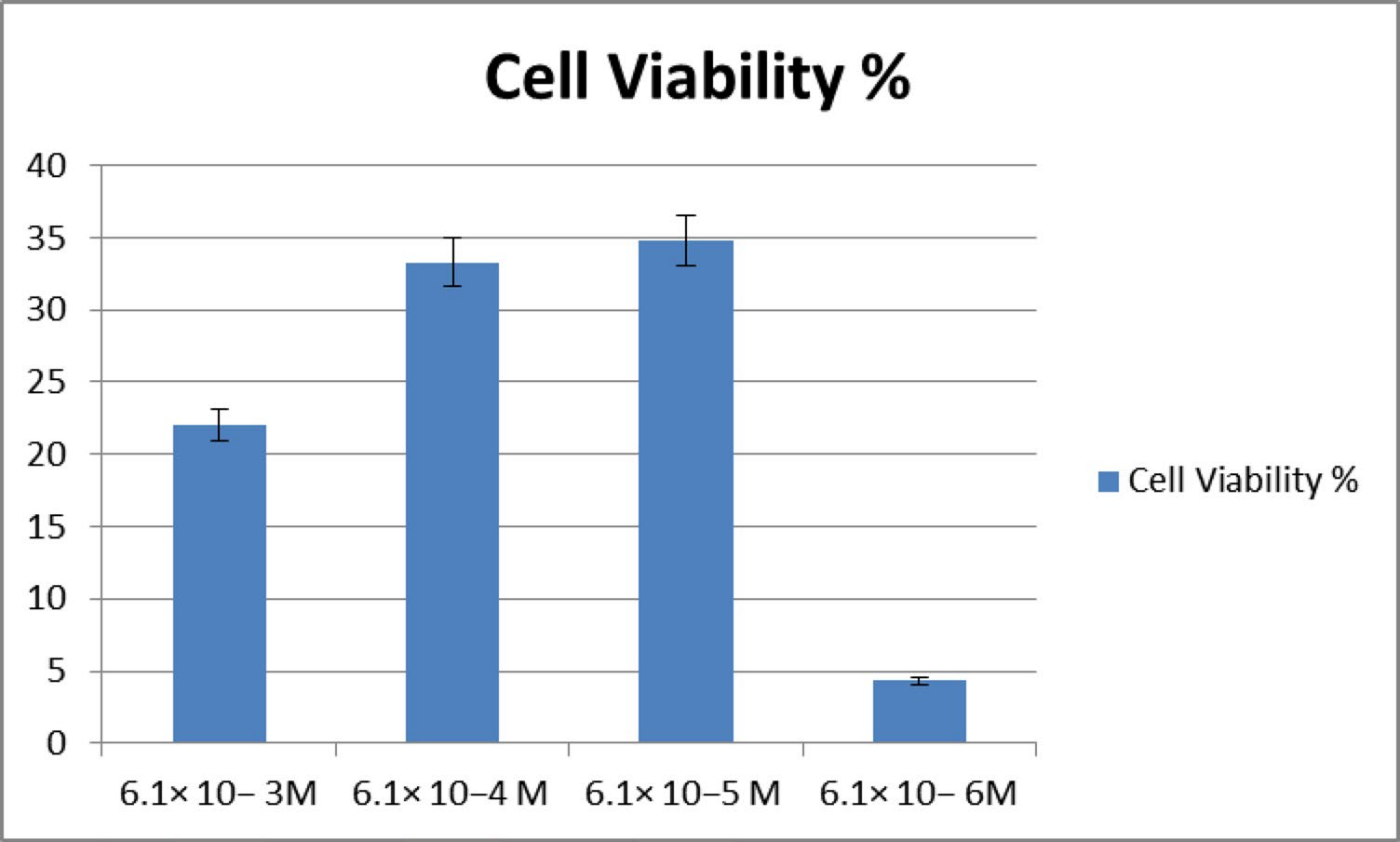
Graphical Representation of Combined Action of MB and Silver NPs on Cancer Cells, The SD values of three independent trials (n = 3) are represented by error bars. The differences between the treatment and control groups were validated through statistical analysis by a one-way ANOVA (p < 0.05).

The information presented shows how different concentrations impact cell viability when methylene blue (MB) and 50 µl of silver nanoparticles (AgNPs) are combined. Cell viability is dramatically decreased to 16.34% at the maximum dose of 6100 µM, suggesting a potent cytotoxic impact. Cell viability increases to 24.08% when the concentration drops 6.1 ×$\:{10}^{-4}$M, indicating a potential reduction in toxicity. Cell viability increases to 49.63% at the lowest concentration of 6.1 µM, and to 36.43% upon further decrease to 61 µM. This pattern points to a dose-response relationship in which higher concentrations cause more toxicity as measured by decreased cell viability, while lower amounts promote increased cell survival. The linear interpolation between the data points where cell viability passes 50% was done using the IC50, or the concentration at which cell viability is decreased by 50%. About 55 µM is the estimated IC50 value. This indicates that MB and AgNPs together diminish cell viability by 50% at this concentration. Since a lower concentration of the combination is needed to significantly reduce cell viability, a lower IC50 implies a stronger action. This data is essential for assessing the combination’s effectiveness and figuring out the ideal concentration for medical or research uses. Graphical representation is shown below. The Fig. 11 displays the effect of combining MB (likely Methylene Blue) with AgNPs (Silver Nanoparticles) at a concentration of 50 µl on cell viability, expressed as a percentage. As the concentration of MB decreases from 6100 µM to 6.1 µM, cell viability increases. At the highest concentration of 6100 µM, cell viability is around 15%, indicating significant cytotoxicity when MB and AgNPs are used together (Fig. 11).

All of the data used in this study has undergone rigorous verification and statistical testing to guarantee accuracy and dependability. To verify treatment-specific effects, control groups were included in every experimental condition. Bacterial inhibition only happened under combined treatment circumstances, as demonstrated by the absence of zones of inhibition in antibacterial assays for negative controls (bacteria without AgNPs, photosensitisers, or light). In photodynamic studies, dark controls (no irradiation) and light-only controls demonstrated no inhibitable activity, proving that photosensitisers’ cytotoxic and antibacterial properties were dependent on light activation.

All quantitative data are presented using the mean ± standard deviation (SD) of three separate replicates (*n* = 3). To show data fluctuation, error bars have been added to all graphical figures. One-way ANOVA was used for statistical analysis, and *p* < 0.05 was deemed significant. The statistical validity and repeatability of experimental data across different trials are guaranteed by these modifications. High-quality photos have been added to all figures, including biological assay figures (Figs. 1, 2, 3, 4, 5, 6, 7, 8, 9, 10 and 11), UV–Vis, SEM, XRD, and FTIR, to improve both visualisation and scientific quality.

## Discussion

With millions of antibiotic-resistant infections and hundreds of related fatalities recorded each year in the United States alone, the growing threat of antibiotic resistance is a major global health problem. Numerous studies into alternate antibacterial techniques, including as photodynamic treatment (PDT), bacteriophage therapy, metallic nanoparticles, and DNA-modifying enzymes, have been spurred by this concerning increase^30^. PDT, which uses light-activated photosensitisers (PS) to produce reactive oxygen species (ROS) that cause microbial and cancer cell death, has become one of the most promising of these. However, the addition of nanomaterials like silver nanoparticles (AgNPs), which have inherent antibacterial and anticancer qualities, outstanding biocompatibility, and the capacity to increase ROS-mediated cytotoxicity, can further increase the therapeutic potential of PDT^32^.

The Pulsed Laser Ablation in Liquid (PLAL) approach, a surfactant-free, ecologically safe technology that guarantees high purity and stability of nanoparticles, was used in the current study to create AgNPs. The generation of well-dispersed spherical AgNPs was confirmed by the UV–Vis spectroscopic examination, which showed a clear surface plasmon resonance (SPR) peak at about 400 nm. While Fourier-transform infrared spectroscopy (FTIR) revealed functional groups on the nanoparticle surface, most likely from stabilising molecules created during the ablation process, X-ray diffraction (XRD) examination confirmed the crystalline character of the nanoparticles. These results validate the reproducibility and dependability of this synthesis method, who showed comparable particle properties under similar PLAL settings.

When combined with the photosensitizer Chlorin e6 (Ce6), the synthesized AgNPs demonstrated a strong synergistic impact in both antibacterial and anticancer evaluations^6,8^. Results from the well diffusion assay indicated that AgNPs effectively suppressed the growth of pathogenic Gram-negative bacteria such as *Salmonella enteritidis* and *Pseudomonas aeruginosa*, producing inhibition zones reaching up to 25 mm. Under photodynamic therapy (PDT) conditions, this antibacterial efficacy was further intensified, as light-activated Ce6–AgNP complexes generated higher levels of reactive oxygen species (ROS), leading to substantial bacterial cell destruction. These findings are consistent with previous studies by^6^, which reported that combining photosensitizers with metallic nanoparticles enhances ROS generation and overall antimicrobial performance. The demonstrated synergism highlights the promise of Ce6–AgNP conjugates as multifunctional therapeutic systems capable of effectively eradicating microbial pathogens while minimizing the emergence of antibiotic resistance.

MCF-7 breast cancer cells were used to test the cytotoxic capability of the synthesised AgNPs, Ce6, and their combination. With an IC²¹ of 138.2 µg/mL, Ce6 alone caused a dose-dependent decrease in cell viability, which is in keeping with its established photosensitising effectiveness in PDT. However, at the highest measured concentration, the combination of Ce6 and AgNPs showed significantly increased cytotoxicity, lowering cell viability to about 24.5%. Improved Ce6 uptake, more ROS production, and AgNPs’ capacity to promote localised electromagnetic field amplification during light exposure are all responsible for this potentiation effect.

The selective cytotoxic behavior of the AgNP–Ce6 complex results from the interplay of passive and active targeting mechanisms. Owing to the Enhanced Permeability and Retention (EPR) effect, nanoparticles within the 20–60 nm range preferentially accumulate in tumor sites because of their permeable vasculature and inefficient lymphatic clearance. Meanwhile, Ce6 activation through controlled light exposure enables precise localization of cytotoxic effects, thereby minimizing unintended damage to healthy, non-irradiated tissues. This combination of passive tumor localization and light-dependent activation provides an ideal therapeutic approach for achieving selective cancer cell eradication^13^.

On a cellular scale, AgNPs promote oxidative stress by overproducing reactive oxygen species (ROS), which disrupt mitochondrial integrity, damage genetic material, and interfere with redox-regulated signaling pathways, ultimately triggering apoptosis. They also bind to thiol-containing enzymes, impairing essential metabolic functions and heightening cytotoxicity. Moreover, their surface plasmon resonance (SPR) enhances the photoexcitation of nearby Ce6 molecules, thereby increasing ROS generation and strengthening the overall photodynamic effect. The evident morphological alterations in MCF-7 cells such as cellular shrinkage and decreased viability further substantiate these underlying mechanistic processes^31^.

This research confirms that AgNPs synthesized through the PLAL method exhibit strong antibacterial and anticancer properties, especially when used in conjunction with Ce6 under photodynamic conditions. The synergistic increase in bacterial inhibition and cancer cell death highlights the potential of these nanocomposites for versatile biomedical use. The incorporation of AgNPs and Ce6 within a photodynamic therapy framework offers a safe, selective, and efficient treatment approach, opening new avenues for the development of advanced nanomedicine-based antimicrobial and anticancer therapies.

## Conclusion

This research effectively demonstrated the production of surfactant-free, high-purity silver nanoparticles (AgNPs) using pulsed laser ablation in liquid (PLAL) an environmentally friendly and contamination-free technique that generated uniformly dispersed nanoparticles with distinct physicochemical features. Detailed characterization using UV–Vis spectroscopy, XRD, FTIR, and SEM verified the synthesis of crystalline, spherical AgNPs with an average diameter of around 25–40 nm and a pronounced surface plasmon resonance (SPR) peak near 410 nm, confirming their stability and uniform dispersion.

The antibacterial studies demonstrated that AgNPs possessed strong, broad-spectrum activity against *Pseudomonas aeruginosa*, *Salmonella enteritidis*, and *Acinetobacter baumannii*, with a marked improvement observed when combined with photosensitizers under photodynamic conditions. The synergistic interaction between AgNPs and either Chlorin e6 (Ce6) or Methylene Blue (MB) under red-light exposure (650–700 nm) led to significantly larger inhibition zones compared to the use of individual agents, indicating that photoactivation greatly enhances bactericidal performance via reactive oxygen species (ROS)-driven pathways. Control experiments confirmed that light irradiation was critical for antimicrobial activity, emphasizing the precision and controllability of the photodynamic response.

Cytotoxicity assessments on the MCF-7 human breast cancer cell line revealed that combining AgNPs with Ce6 and MB produced strong, dose-dependent antiproliferative effects. The observed IC₅₀ values—138.2 µM for Ce6 and 4.92 µM for MB—and the substantial decrease in cell viability (~ 24.5%) at higher combined concentrations indicate a pronounced enhancement of photodynamic efficacy through nanoparticle–photosensitizer interaction. These results imply that AgNPs not only promote photosensitizer internalization and cellular uptake but also intensify reactive oxygen species (ROS) generation via plasmonic coupling, thereby achieving effective cancer cell destruction with precise spatiotemporal regulation through light activation.

Overall, these findings demonstrate that PLAL-synthesized AgNPs, when combined with photosensitizers like Ce6 and MB, display exceptional synergistic antibacterial and anticancer properties under photodynamic conditions in agreement with recent advancements in nanoparticle-based biomedical research^2,15,4^. The dual-functional performance of this nanocomposite system highlights its potential as an advanced, resistance-free therapeutic approach for both infectious disease control and cancer therapy. Future research should aim to further clarify the molecular pathways responsible for ROS generation, refine nanoparticle surface engineering to enhance biocompatibility, and extend these investigations to in-vivo studies to validate safety and clinical applicability. 

## Data Availability

The data that support the findings of this study is available on request from the corresponding author. The data is not publicly available due to privacy and ethical restrictions.
